# Association between serum lipid levels and the risk of diabetic nephropathy: a meta-analysis

**DOI:** 10.3389/fendo.2026.1783038

**Published:** 2026-04-22

**Authors:** Linli Cai, Xingyuan Li, Qing Yang, Fang Liu

**Affiliations:** 1 Department of Nephrology, West China Hospital of Sichuan University, Chengdu, China; 2 Laboratory of Diabetic Kidney Disease, Centre of Diabetes and Metabolism Research, West China Hospital of Sichuan University, Chengdu, China

**Keywords:** diabetic nephropathy, high-density lipoprotein cholesterol, lipid, low-density lipoprotein cholesterol, total cholesterol, triglycerides

## Abstract

**Background:**

Observational studies on the association between specific lipid parameters—such as triglycerides (TG), total cholesterol (TC), high-density lipoprotein cholesterol (HDL-C), and low-density lipoprotein cholesterol (LDL-C)—and the risk of diabetic nephropathy (DN) have yielded inconsistent and sometimes controversial conclusions.

**Methods:**

This study systematically searched relevant literature in PubMed, Embase, Web of Science, and the Cochrane Library up to October 2025. Statistical analysis was performed using Review Manager 5.4.1 software. The pooled odds ratio and its 95% confidence interval were calculated using a random-effects model. Heterogeneity was assessed using the chi-square test and I² statistic, and publication bias was assessed using funnel plots and Egger regression tests. The effect of publication bias was analyzed using the trim-and-fill method, and the robustness of the results was examined through sensitivity analysis. Evidence for each outcome was evaluated and graded according to GRADE.

**Results:**

A total of 15 studies were included in the meta-analysis. The results showed that higher serum TG levels were significantly associated with an increased risk of DN (OR = 1.17, 95% CI: 1.11–1.23, P < 0.00001). Higher TC levels also indicated a slight increased risk (OR = 1.06, 95% CI: 1.01–1.11, P = 0.01). HDL-C showed a protective effect (OR = 0.86, 95% CI: 0.81–0.92, P < 0.00001). No significant association was found between LDL-C and the risk of DN. Egger’s test suggested publication bias for TG and HDL-C, but the magnitude of strength did not change direction after trimming and filling. Sensitivity analysis showed that the above findings were robust. Regarding the GRADE rating, all outcomes were rated as very low-quality evidence.

**Conclusion:**

Elevated serum TG levels are a risk factor for DN, while HDL-C shows a protective effect. While TC showed a positive correlation, the effect was weak; LDL-C did not show a significant association. Future research should focus on prospective cohort designs to validate causal associations and explore the potential value of lipid-lowering therapy in the primary prevention of DN.

## Introduction

Diabetic nephropathy (DN), one of the most serious and common microvascular complications of diabetes, has become a leading cause of end-stage renal disease worldwide (1). It not only significantly increases patient mortality and cardiovascular event risk but also imposes a heavy socioeconomic burden (2). With the continued rise in global diabetes prevalence, the prevention and treatment of DN have become an important public health issue (3). In the development of DN, multiple metabolic abnormalities interact, among which lipid metabolism disorders, as a common complication of diabetes, are increasingly attracting scholarly attention (4, 5). Previous studies suggest that dyslipidemia may participate in the pathophysiological process of DN by promoting inflammatory responses, oxidative stress, and endothelial dysfunction, thereby exacerbating glomerular sclerosis and tubulointerstitial fibrosis (6, 7). However, existing observational studies on the association between specific lipid parameters—such as triglycerides (TG), total cholesterol (TC), high-density lipoprotein cholesterol (HDL-C), and low-density lipoprotein cholesterol (LDL-C)—and the risk of DN have yielded inconsistent and sometimes controversial conclusions (8–10). Some studies have reported significant positive or negative correlations (11, 12), while others have not found a clear association (13, 14). This inconsistency may stem from heterogeneity in the study populations, sample size limitations, inadequate adjustment for confounding factors, and differences in lipid measurement methods as well as the definition criteria for DN. Therefore, a systematic meta-analysis is urgently needed to comprehensively assess the existing evidence and clarify the predictive value of serum lipid levels for the risk of DN.

The mechanisms by which dyslipidemia plays a complex and multifaceted role in diabetic complications are diverse. From a pathophysiological perspective, hypertriglyceridemia can directly impair glomerular filtration barrier function by increasing renal lipid deposition, inducing oxidative stress, and triggering inflammatory responses (15). Elevated LDL-C levels may weaken its inherent anti-inflammatory, antioxidant, and cholesterol reverse transport capabilities, leading to vascular endothelial dysfunction and renal microcirculatory disturbances (16). Furthermore, lipid metabolism disorders intertwine with insulin resistance and chronic inflammation, creating a vicious cycle that further accelerates the progression of DN (17). Although statins are highly effective in lowering LDL-C and cardiovascular risk, their exact protective effect against DN remains controversial (18). This suggests that we may need to move beyond the traditional LDL-C perspective and re-examine the unique value of other lipid parameters in the development of DN.

Currently, there are significant gaps and limitations in the evidence regarding lipid parameters and the risk of DN. Most existing studies have limited sample sizes, short follow-up periods, and lack heterogeneity analyses across different populations (e.g., gender, age, and duration of diabetes). More importantly, previous studies have largely focused on single lipid parameters, lacking a systematic assessment of the combined effects of multiple lipid indicators. This fragmented evidence landscape limits clinicians’ ability to optimize lipid management strategies for DN. Therefore, integrating existing evidence through rigorous systematic reviews and meta-analyses, quantifying the strength of the association between different lipid parameters and the risk of DN, and exploring the sources of heterogeneity are not only of significant theoretical importance but also provide evidence-based support for clinical practice. This study aims to fill this evidence gap and provide new insights for the early prevention and precise intervention of DN.

## Methods

### Literature search

This meta-analysis was conducted in accordance with the PRISMA 2020 statement (19) and was registered in PROSPERO (CRD420251231149). To identify relevant studies assessing the association between serum lipid levels and the risk of DN, a systematic literature search was performed in PubMed, Embase, Web of Science, and the Cochrane Library up to October 2025. The search terms used were: “Triglycerides”, “Low-Density Lipoprotein Cholesterol”, “High-Density Lipoprotein Cholesterol”, and “Total Cholesterol”. To ensure completeness, the bibliographies of all included articles were manually screened. Two investigators independently performed article selection and study quality assessment, resolving discrepancies by discussion. **Supplementary Table 1** presents the detailed search strategies.

### Inclusion and exclusion criteria

Inclusion criteria:

P: High-risk individuals for DN, regardless of whether they have type 2 diabetes.

E: Highest TG, TC, HDL-C, and LDL-C (the specific high and low dividing lines depend on each individual study).

C: Lowest TG, TC, HDL-C, and LDL-C.

O: The incidence of DN.

S: Study design was cohort or case-control.

Exclusion criteria: Study protocols, unpublished studies, non-original publications such as letters, abstracts, comments, corrections, and replies. Additionally, single-arm studies, review articles, and studies lacking sufficient data—specifically those from which hazard ratios, odds ratios (ORs) and corresponding 95% confidence intervals (CIs) could not be directly obtained or calculated—were also excluded.

### Data abstraction

Two investigators independently extracted data. A third author resolved any disagreements. The following information was collected from each eligible study: first author’s name, year of publication, geographic location, study design, population characteristics, sample size, patient age and sex, and the OR of the correlation between serum lipid levels and the risk of developing DN (OR values from multivariate adjusted models (Cox or Logistic regression) were extracted and merged first). If essential data were unavailable or incomplete, corresponding authors were contacted for further clarification or supplementary information.

### Quality evaluation

The quality of the included cohort and case-control studies was determined based on the Newcastle–Ottawa Scale criteria (NOS) (20), with scores ranging from 7 to 9 indicating high quality (21). Quality assessments were performed independently by two reviewers, and any disagreements in their judgment were addressed and settled by discussion.

### Statistical analysis

Using Review Manager (v5.4.1), we performed the analysis, employing ORs for data synthesis. Each metric was presented with 95% CIs using a random-effects model. Chi-squared (χ^2^) test (Cochran’s *Q*) and the inconsistency index (*I*^2^) were used to evaluate the heterogeneity of each outcome (22, 23). Thresholds for high heterogeneity were defined as P < 0.1 and I² > 50%. For outcomes that included more than two studies, a sensitivity analysis was performed to evaluate each study’s impact on the overall estimate. In addition, this study conducted subgroup analyses on results with ≥10 studies based on the study design to explore the stability of the results and potential sources of heterogeneity. Potential publication bias was assessed using Egger’s regression test (24) in Stata 15.1 (Stata Corp, College Station, Texas, USA) for outcomes with 10 or more included studies. A threshold of P < 0.05 was used to identify significant evidence of publication bias. For results with publication bias, the trim-and-fill method was used to assess its impact on the results. Additionally, according to GRADE, each outcome’s evidence was evaluated and graded as “high”, “moderate”, “low”, or “very low” quality (25).

## Results

### Literature retrieval, study characteristics, and baseline

**Figure 1:**
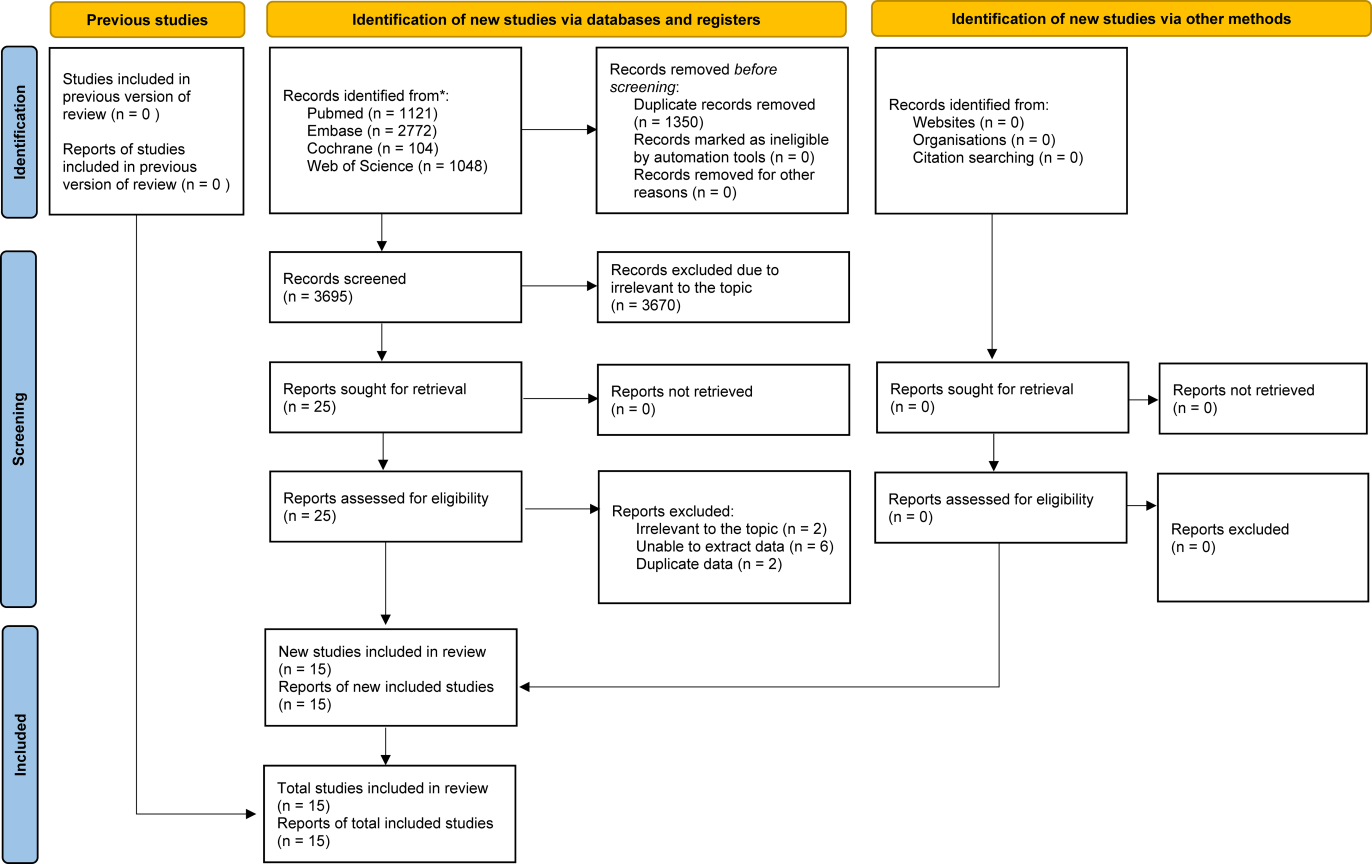
Flowchart of the systematic search and selection process.

The process of literature identification and selection is illustrated in **Figure 1**. A comprehensive search across PubMed (n = 1121), Embase (n = 2772), Web of Science (n = 1048), and the Cochrane Library (n = 104) initially yielded a total of 5045 records. After duplicate entries were removed, 3695 unique titles and abstracts were screened for relevance. In the final analysis, 15 studies were deemed eligible and included in the meta-analysis (11–14, 26–36). **Table 1** summarizes the key features and methodological quality of each eligible study. The included literature was published between 2012 and 2025.

**Table 1:** Characteristics and quality assessment of included studies.

Study	Country	Study design	Population	No. of patients	Gender		Mean/median age	NOS score	Diagnostic criteria for diabetic nephropathy	Adjustment factors
					Male	Female				
Aboelnasr 2020	Egypt	Case-control	Patients Newly Diagnosed with Type-2 Diabetes Mellitus	154	57	97	49.1	7	UACR is increased or if eGFR is reduced in the absence of signs or symptoms of other primary causes of kidney damage.	Smoking history
Ahn 2014	Korea	Case-control	Patients aged >30 years	4652	2380	2272	49.2	7	Diabetes with the presence of albuminuria or impaired GFR.	Age, sex, waist circumference, alcohol consumption, smoking, exercise
Al-Shahrani 2025	Saudi Arabia	Case-control	Type 2 diabetes patients	419	240	179	58.5	7	The American Diabetes Association criterion was used to identify cases with microalbuminuria, macroalbuminuria, and end−stage renal disease.	Age, sex, diabetes duration
Dejenie 2023	Ethiopia	Case-control	Patients with type 2 diabetes mellitus	140	74	66	45	8	NA	Age, BMI, SBP, DBP, diabetes duration
Farah 2021	Jordan	Case-control	Patients with type 2 diabetes mellitus	1398	573	825	NA	7	DKD was defined as a reduced eGFR, <60 mL/min/ 1.73 m2, and/or increased urinary albumin excretion, ≥30 mg/g creatinine that persisted for ≥3 months in the presence of longstanding diabetes and exclusion of other causes of CKD.	Sex, age, smoking, hypertension, stroke, diabetes duration
Gong 2021	China	Cohort	Patients Newly Diagnosed with Type-2 Diabetes Mellitus	11142	4148	6994	58.2	7	Incident DKD was defined by an eGFR<60 mL/min/1.73 m2 at the follow-up visit.	Sex, age, smoking, diabetes, obesity
Liu 2022	China	Case-control	Patients with type 2 diabetes mellitus	791	475	316	61	7	In this study, albumin- to- creatinine ratio(ACR) was used to define DKD; for a better definition of DKD in our population, repeated microalbuminuria measurements have been performed and analysed, and patients with ACR ≥30 mg/g were defined as patients with DKD.	Age, SBP, waist circumference
Morton 2012	Australia	Cohort	Patients with type 2 diabetes mellitus	11140	6400	4740	65.9	7	Total renal events were defined as the development of new microalbuminuria (urinary ACR 30–300 mg/mg), new macroalbuminuria (urinary ACRof.300mg/mg), a doubling of creatinine to at least 200 mmol/L, the need for renal replacement therapy, or death as a result of renal disease.	NA
Russo 2016	Italy	Cohort	Patients with type 2 diabetes mellitus	15362	9013	6349	64	7	The primary outcomes were 1)eGFR,60 mL/min/1.73 m2; 2) albuminuria; 3) eGFR ,60 mL/min/1.73 m2 and albuminuria; and 4)eGFR reduced .30%. The occurrence of prespecified end points was evaluated on a yearly basis over the 4-year study period.	eGFR, albuminuria
Wan 2024	China	Case-control	Patients with type 2 diabetes mellitus	80360	30339	50021	61.4	7	DKD was defined as having albuminuria, a decreased GFR, or both.	Age, sex, ethnic, diabetes duration, BMI
Wang Huabin 2025	China	Cohort	Patients with type 2 diabetes and no baseline evidence of DKD	3040	1877	1163	58.04	7	DKD was defined as ACR ≥ 30 mg/g or a decrease in eGFR < 60 mL/min/1.73 m² during follow-up. eGFR was calculated using the Xiangya equation.	NA
Wang Jia 2025	China	Case-control	Patients with primary types 1 and 2 diabetes	15431	7904	7527	64	7	In accordance with the International Classification of Diseases (ICD9 or ICD10 codes)	NA
Xiang 2024	China	Case-control	Patients with type 2 diabetes mellitus	1313	772	541	59.2	7	DKD was defined as eGFR<60 ml/min/1.73 m2 and/or ACR>30 mg/g for at least three months caused by diabetes.	Sex
Yang 2019	China	Case-control	Patients with type 2 diabetes mellitus	3698	1604	2094	66.8	7	DKD was defined as either albuminuria or an estimated glomerular filtration rate (eGFR) of < 60 (ml·min− 1(173m2)− 1) according to the Modification of Diet in Renal Disease	Sex
Yang 2022	China	Case-control	Patients with type 2 diabetes mellitus	706	438	268	56.84	7	DN was divided into three stages: non-albuminuria (NAU) with UAER < 20μg/min, microalbuminuria (MAU) with 20≤UAER≤200μg/min, and clinical albuminuria (CAU) with UAER>200μg/ min	Hypertension, HbA1c, BMI

### Association between serum TG levels and DN incidence

**Figure 2:**
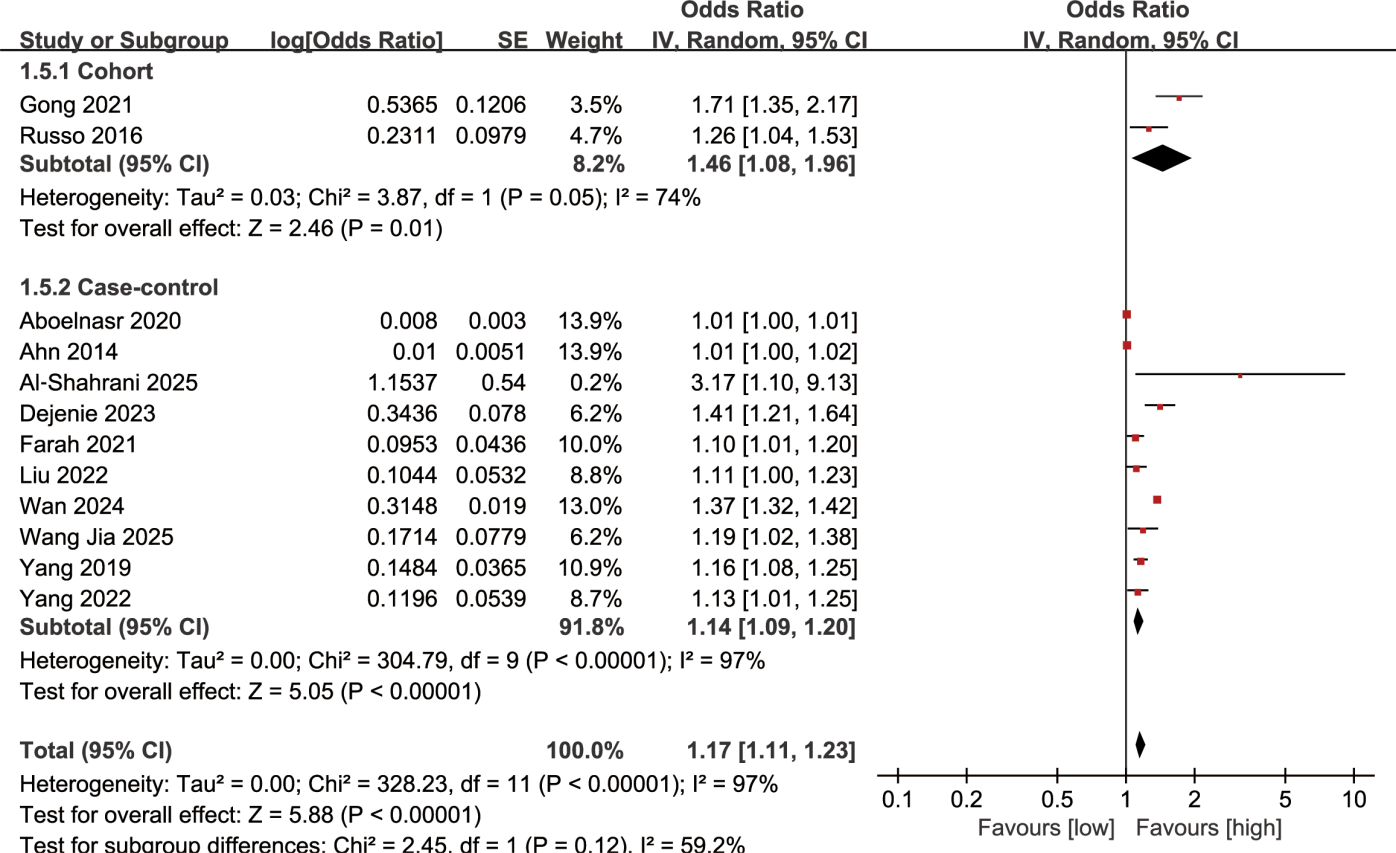
Forest plots of the association between serum TG levels and DN incidence.

Association between serum TG levels and DN incidence was synthesized from 12 studies. The meta-analysis indicated that higher serum TG levels were significantly linked to an increased risk of DN (OR: 1.17; 95% CI: 1.11, 1.23; *P* <0.00001), with substantial heterogeneity (*I*^2^ = 97%, *P* <0.00001) (**Figure 2**). Subgroup analysis based on the study design revealed a significant correlation between serum TG levels and the incidence of DN in both cohort (OR: 1.46; 95% CI: 1.08, 1.96; *P* = 0.01) and case-control studies (OR: 1.14; 95% CI: 1.09, 1.20; *P* <0.00001), with heterogeneity decreasing to some extent in the cohort studies (*I*^2^ = 74%) (**Figure 2**).

### Association between serum TC levels and DN incidence

**Figure 3:**
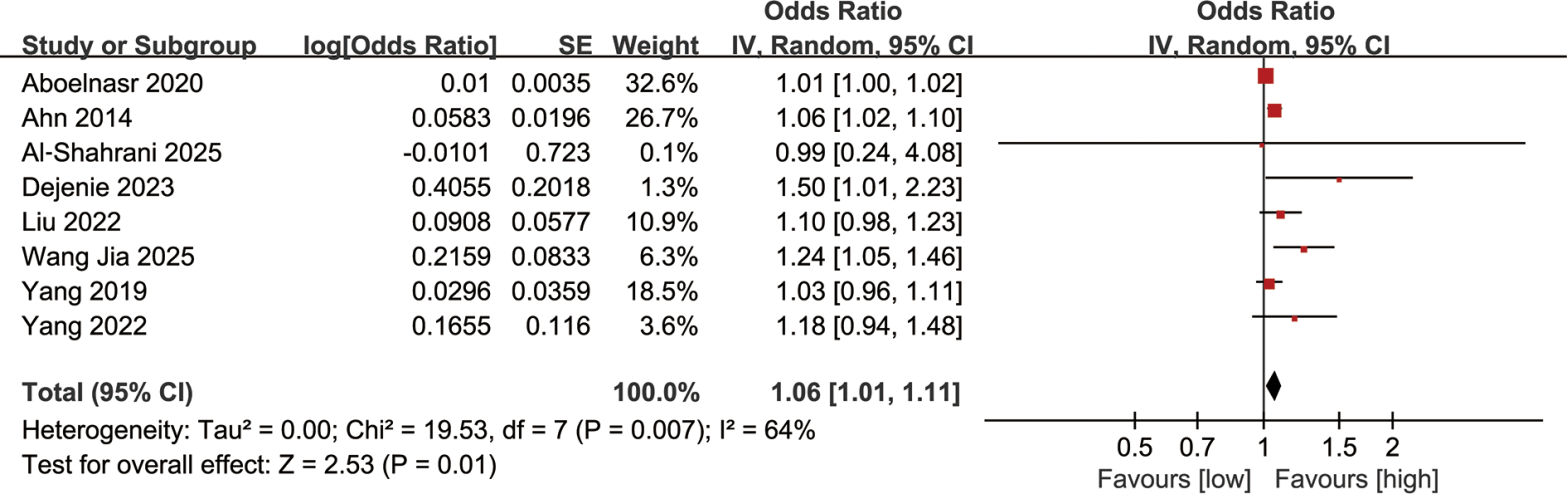
Forest plots of the association between serum TC levels and DN incidence.

Association between serum TC levels and DN incidence was synthesized from 8 studies. The meta-analysis demonstrated that a higher serum TC level was significantly linked to an increased risk of DN (OR: 1.06; 95% CI: 1.01, 1.11; *P* = 0.01), with substantial heterogeneity (*I*^2^ = 64%, *P* = 0.007) (**Figure 3**).

### Association between serum HDL-C levels and DN incidence

**Figure 4:**
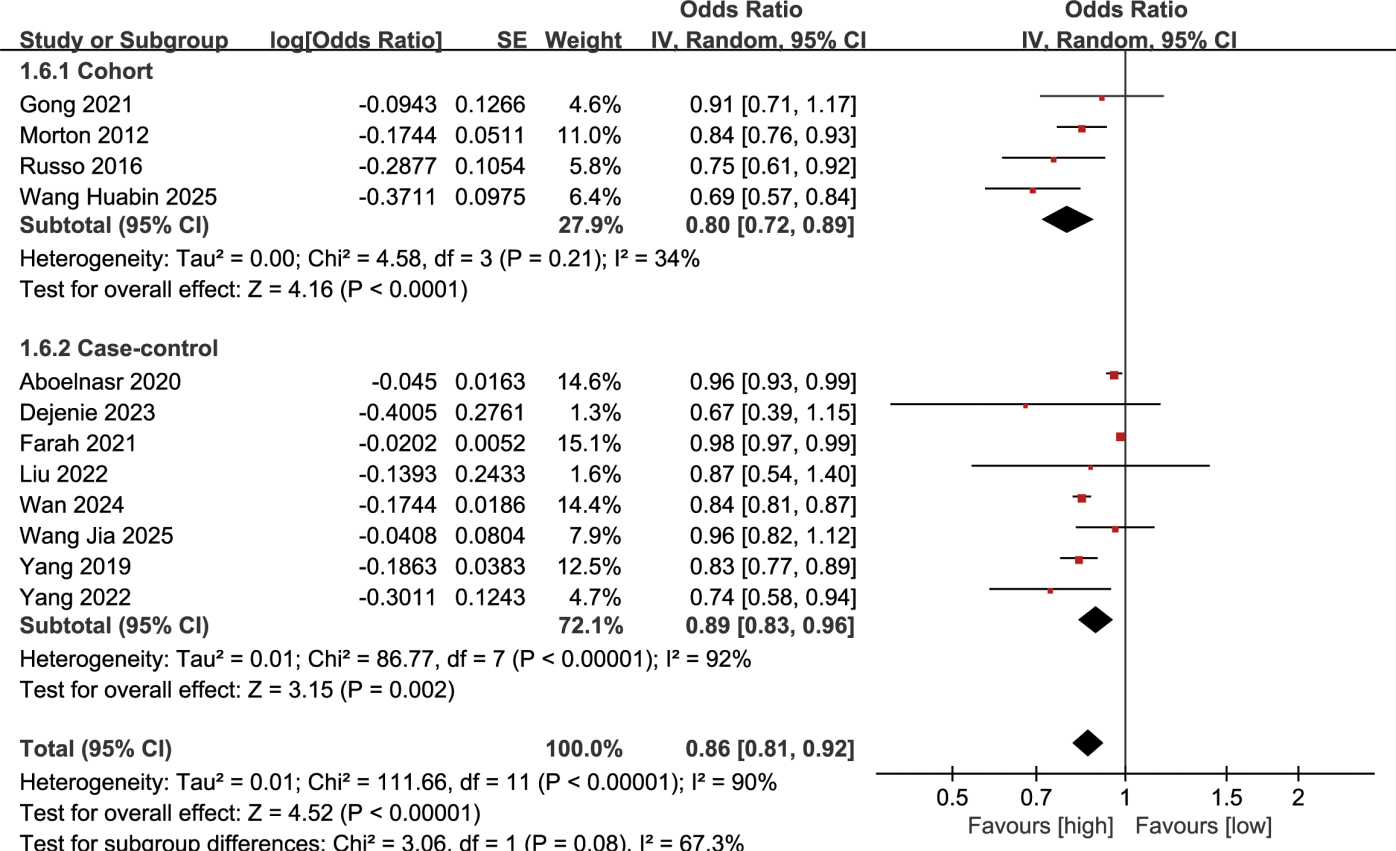
Forest plots of the association between serum HDL-C levels and DN incidence.

Association between serum HDL-C levels and DN incidence was synthesized from 12 studies. The meta-analysis demonstrated that a higher serum HDL-C level was significantly linked to a decreased risk of DN (OR: 0.86; 95% CI: 0.81, 0.92; *P* <0.00001), with substantial heterogeneity (*I*^2^ = 90%, *P* <0.00001) (**Figure 4**). Subgroup analysis based on the study design revealed a significant correlation between serum HDL-C levels and the incidence of DN in both cohort (OR: 0.80; 95% CI: 0.72, 0.89; *P* <0.0001) and case-control studies (OR: 0.89; 95% CI: 0.83, 0.96; *P* <0.00001), with heterogeneity decreasing to some extent in the cohort studies (*I*^2^ = 34%) (**Figure 4**).

### Association between serum LDL-C levels and DN incidence

**Figure 5:**
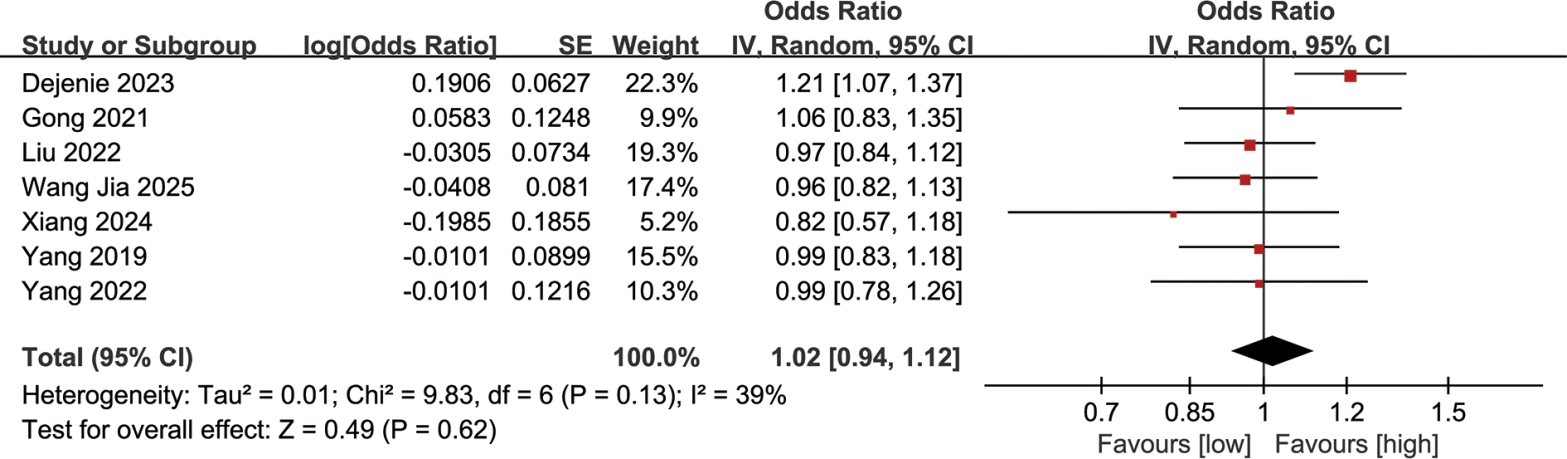
Forest plots of the association between serum LDL-C levels and DN incidence.

Association between serum LDL-C levels and DN incidence was synthesized from 7 studies. The meta-analysis results demonstrated no significant correlation between serum LDL-C levels and the incidence of DN (OR: 1.02; 95% CI: 0.94, 1.12; *P* = 0.62), and no significant heterogeneity was observed (*I*^2^ = 39%, *P* = 0.13) (**Figure 5**).

### Publication bias

**Figure 6:**
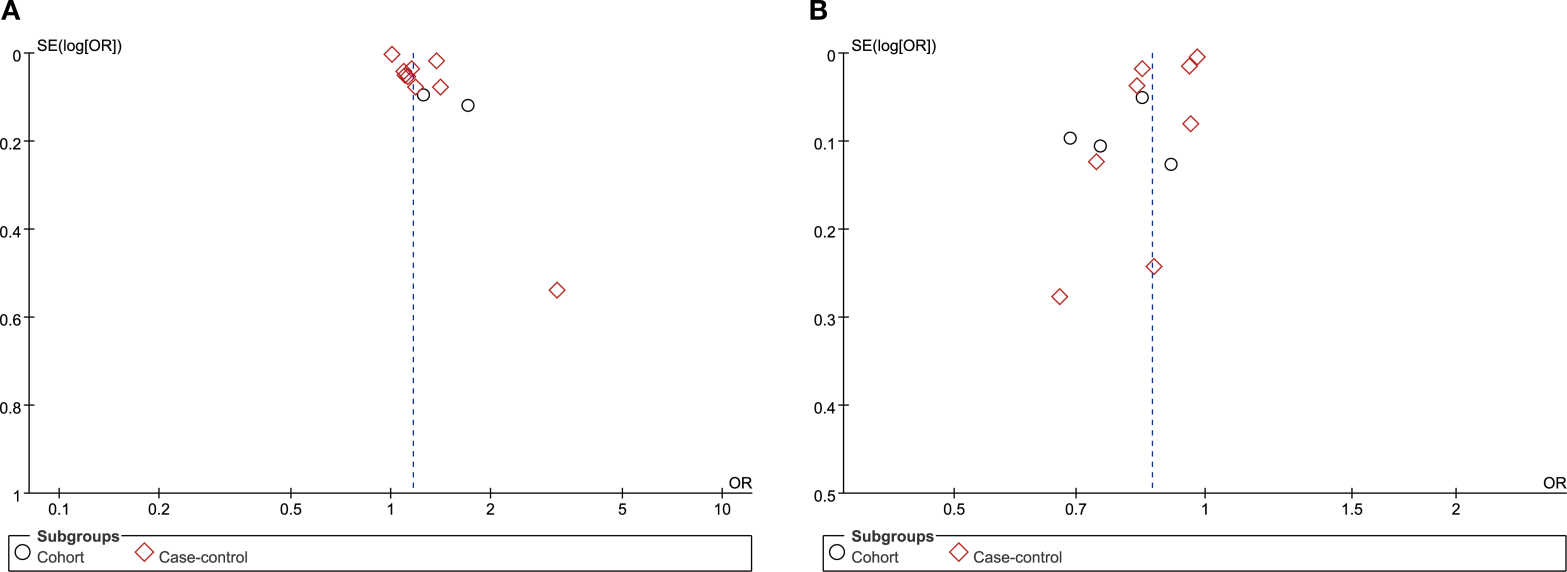
Funnel plot of **(A)** TG and **(B)** HDL-C.

**Figure 7:**
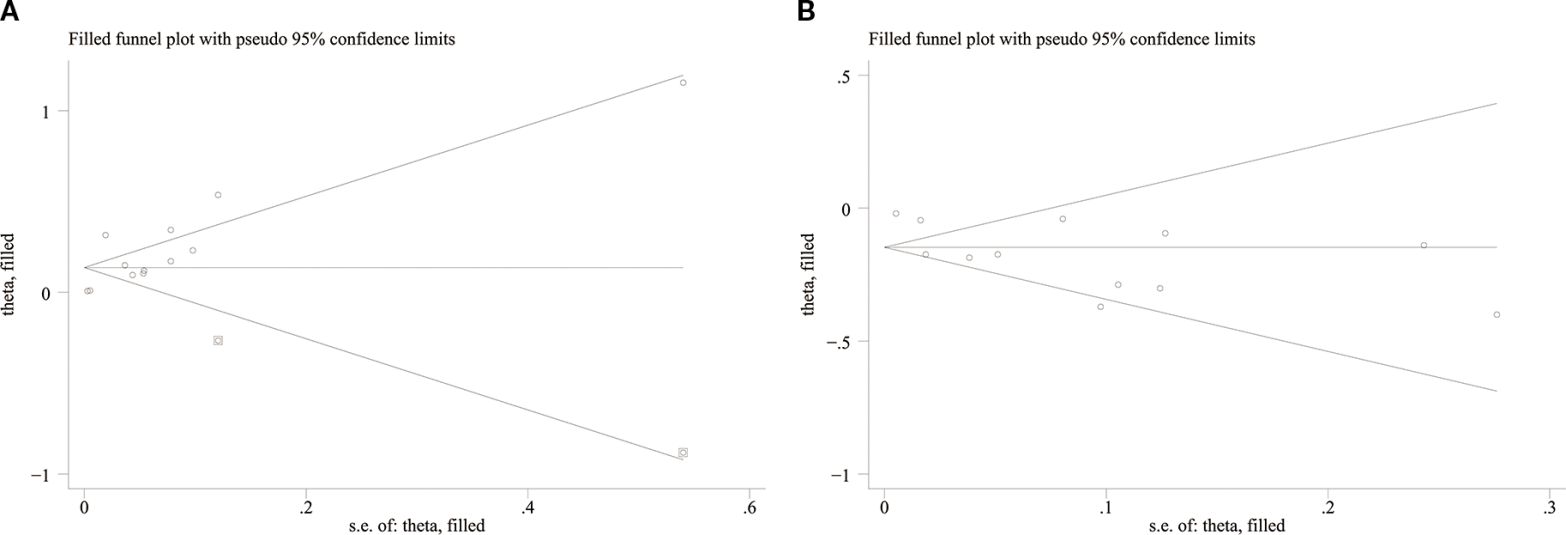
Trimming and filling funnel plot of **(A)** TG and **(B)** HDL-C.

Potential publication bias regarding the associations between serum TG and HDL-C levels and DN incidence was assessed using Egger’s regression test and funnel plot analysis. The results indicated the presence of significant publication bias for TG (Egger’s test P = 0.019, **Figure 6A**) and HDL-C (Egger’s test P = 0.013, **Figure 6B**). The impact of publication bias on TG and HDL-C was assessed using the trim-and-fill method. The results indicated that the significant correlation between TG (OR: 1.15; 95% CI: 1.09, 1.21) (**Figure 7A**) and HDL-C (OR: 0.86; 95% CI: 0.81, 0.92) (**Figure 7B**) and the risk of DN did not change after trimming and filling, suggesting that TG and HDL-C were not affected by publication bias.

### Sensitivity analysis

**Figure 8:**
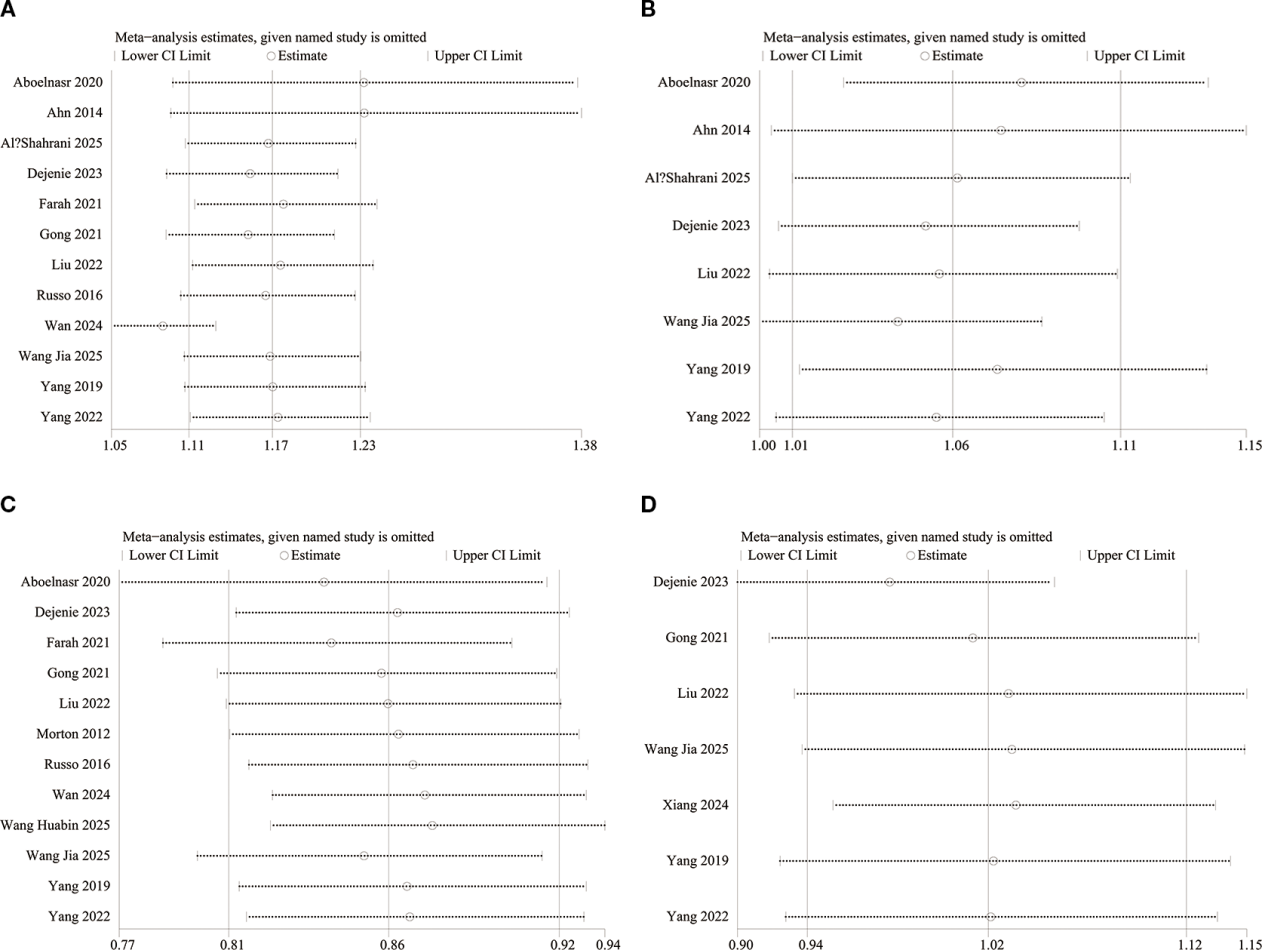
Sensitivity analysis of the association between **(A)** TG, **(B)** TC, **(C)** HDL-C, **(D)** LDL-C and the risk of DN.

We performed a sensitivity analysis to analyze each study’s effect on the overall OR for the associations between serum TG, TC, HDL-C, and LDL-C levels and DN incidence using a leave-one-out approach. The overall ORs for TG (**Figure 8A**), TC (**Figure 8B**), HDL-C (**Figure 8C**), and LDL-C (**Figure 8D**) remained consistent following the sequential exclusion of individual studies.

### GRADE rating

Regarding evidence recommendation, very low-quality evidence was assigned to TG, TC, HDL-C and LDL-C (**Table 2**). The level of evidence was downgraded to ‘very low’ primarily due to limitations in the retrospective study design, significant heterogeneity among outcomes, overly wide confidence intervals for some outcomes and significant publication bias. These factors collectively reduced our confidence in the effect estimates.

**Table 2:** GRADE rating of outcomes.

Types of serum lipid	No. of studies	OR	95%CI	*I*^2^; P value	Risk of bias	Inconsistency	Indirectness	Imprecision	Publication bias	Plausible confounding	Magnitude of effect	Dose-response gradient	GRADE
TG	12	1.17	1.11, 1.23	97%; P<0.00001	No serious risk	Serious inconsistency	No serious indirectness	No serious imprecision	Strongly suspected	Would not reduce effect	No	No	Very low
TC	8	1.06	1.01, 1.11	64%; P=0.007	No serious risk	Serious inconsistency	No serious indirectness	Serious imprecision	NA	Would not reduce effect	No	No	Very low
HDL-C	12	0.86	0.81, 0.92	90%; P<0.00001	No serious risk	Serious inconsistency	No serious indirectness	No serious imprecision	Strongly suspected	Would not reduce effect	No	No	Very low
LDL-C	7	1.02	0.94, 1.12	39%; P=0.13	No serious risk	No serious inconsistency	No serious indirectness	Serious imprecision	NA	Would not reduce effect	No	No	Very low

## Discussion

This meta-analysis of 15 observational studies showed that higher serum triglyceride and TC levels were significantly associated with an increased risk of DN, with pooled OR values of 1.17 and 1.06, respectively. Higher HDL-C levels, however, showed a protective effect, with an OR of 0.86. No statistically significant association was found between LDL-C and DN risk. These findings suggest that different types of lipid metabolism disorders may play different roles in the development of DN, requiring differentiated treatment of different lipid indicators in clinical assessment and intervention. The strong positive correlation between TG and DN risk is consistent with most previous studies, and the underlying biological mechanism may involve inflammatory responses, oxidative stress, and glomerular lipotoxicity triggered by the deposition of triglyceride-rich lipoproteins in the kidneys, leading to glomerular filtration barrier damage and mesangial matrix proliferation (15, 37). The protective effect of HDL-C may be related to its involvement in cholesterol reverse transport, anti-inflammatory properties, and antioxidant effects, which help maintain the endothelial stability of renal microvessels (11, 12). The weak positive correlation between TC and overall cholesterol suggests that overall cholesterol load may have some impact on renal function, but the effect is relatively weak. Therefore, at the individual patient level, such minor changes in relative risk need to be assessed in conjunction with absolute risk to comprehensively evaluate their importance and avoid over-interpretation. The lack of correlation between LDL cholesterol and the risk of DN may be related to the widespread use of statins among study participants, differences in LDL cholesterol subtype distribution, or residual confounding factors between studies.

Regarding heterogeneity analysis, this study found high heterogeneity in the association analysis of TG, TC, and HDL-C with DN, with I² statistics reaching 97%, 64%, and 90%, respectively. This significant heterogeneity may stem from differences in included studies across various aspects, including population characteristics, study design, lipid measurement methods, definition criteria for DN, and the degree of adjustment for potential confounding factors. To identify potential sources of heterogeneity, we conducted a sensitivity analysis, using a method of eliminating individual studies one by one for validation. The results showed that the pooled odds ratio did not change in direction, indicating that the main conclusions of this study have good robustness. Nevertheless, the existence of high heterogeneity suggests that we should interpret pooled effect sizes with caution. Furthermore, although the trimming and supplementation method did not change the direction of the association, the presence of publication bias further weakened our confidence in the accuracy of the effect size point estimates and constituted one of the reasons for the downgrade of the GRADE rating. Future studies will require more rigorously designed and methodologically consistent prospective studies to further validate these associations, and meta-analyses of individual participant data to explore the exact causes of heterogeneity more deeply.

It must be recognized that this study’s analysis treated DN as a single clinical outcome, potentially overlooking its inherent phenotypic heterogeneity. Increasing evidence suggests that diabetic nephropathy encompasses diverse clinicopathological phenotypes, such as the albumin-dominant phenotype and the non-albuminuria phenotype dominated by decreased eGFR, which may differ in risk factors, disease progression trajectories, and prognoses (38). The original studies included in this meta-analysis generally employed traditional composite definitions based on albuminuria or decreased eGFR, failing to provide detailed data stratified by different phenotypes. Therefore, we were unable to explore whether specific lipid parameters were more strongly associated with a particular DN phenotype. This inadequate resolution of phenotypic heterogeneity may be a significant reason for the extremely high statistical heterogeneity observed in this study. Furthermore, dyslipidemia may exacerbate glomerular or tubulointerstitial damage through different mechanisms, which are collectively considered in traditional clinical endpoints. Therefore, future prospective studies need to focus on collecting and reporting phenotype-based endpoint data to more accurately reveal the role of dyslipidemia in different progression pathways of diabetic nephropathy.

Placing these findings within a broader academic context, they largely align with previous observational studies and meta-analyses exploring the relationship between blood lipids and DN, particularly in confirming a significant link between TG and HDL-C (11, 12, 18, 26, 36). However, by incorporating more recent and numerous studies, this study provides a more precise estimate of the effect and strengthens the evidence. Furthermore, our finding of no significant association with LDL-C differs from some traditional views that emphasize LDL-C as a risk factor for DN. This suggests that in predicting the risk of DN, we may need to focus more on indicators such as TG and HDL-C, rather than solely on LDL-C. This difference may also reflect specific responses across different populations, at different disease stages, or under different treatment contexts.

From the perspective of underlying biological mechanisms, the pathways by which dyslipidemia participates in the development of DN are multifaceted. Elevated TG levels may lead to lipid accumulation in the kidneys, triggering lipotoxicity and activating multiple inflammatory pathways and fibrotic processes (39). HDL-C may protect kidney cells from damage through its antioxidant and anti-inflammatory properties (40), as well as its ability to promote cholesterol efflux (41, 42). Elevated TC, as an indicator of overall lipid load, may reflect the overall level of atherogenic lipoproteins (7), thereby indirectly affecting kidney function. The link between LDL-C and the risk of DN is not significant, possibly suggesting that other lipid metabolism disorders may be more critical in the diabetic state, or that the widespread use of statins may have obscured this association. Future basic research will need to further elucidate the specific mechanisms of action of these lipid parameters at the kidney cellular level and their interactions with diabetes-related metabolic disorders (such as insulin resistance).

Beyond direct metabolic toxicity, the evidence integrated in this review fails to adequately capture the crucial mediating role of chronic low-grade inflammation and innate immune activation associated with lipid disorders, particularly those in insulin resistance, in kidney injury. High triglycerides and low HDL-C phenotypes are closely associated with elevated circulating pro-inflammatory cytokines, endothelial dysfunction, and microvascular damage, collectively constituting the core pathological background of metabolic inflammation driving the progression of diabetic nephropathy. Studies have indicated that even simple systemic inflammatory markers such as the neutrophil-lymphocyte ratio (NLR) can effectively reflect this residual inflammatory risk and are independently associated with subclinical vascular damage in individuals with metabolic disorders (43). However, the original literature included in this study generally lacks simultaneous measurement and correction of inflammatory markers, limiting our ability to analyze the relative contributions of lipid metabolism pathways and inflammatory pathways in the development of diabetic nephropathy, representing a significant limitation of the current evidence framework. Future research needs to integrate metabolic and inflammatory indicators to more comprehensively assess the overall risk of diabetic nephropathy.

The results of this study are based on traditional clinical endpoints for DN. While these endpoints are clinically significant, they may not be sensitive enough to detect earlier kidney damage. Recent evidence suggests that metabolic and lipid disorders can directly induce stress and damage to renal tubular epithelial cells, a process that may occur much earlier than a decrease in glomerular filtration rate or the appearance of overt proteinuria, and can be detected by specific urinary biomarkers (44). Therefore, the association between lipid parameters and clinical DN revealed in this meta-analysis may only reflect the role of lipid abnormalities in a later stage of the kidney injury continuum; their potential impact on early subclinical tubular damage may be underestimated by the current evidence framework based on traditional endpoints. This suggests that lipid abnormalities may be involved in the progression of diabetic nephropathy earlier than currently observed in clinical practice, and future research needs to incorporate novel renal tubular injury biomarkers to more comprehensively assess the value of lipid management in the early prevention of diabetic nephropathy.

The results of this study have clear clinical significance. First, they suggest that in the daily management of diabetic patients, in addition to routine monitoring of blood glucose and LDL cholesterol, TG and HDL cholesterol should also be considered important indicators for assessing the risk of DN. Diabetic patients with high TG and/or low HDL cholesterol should be considered a high-risk group for DN, requiring enhanced monitoring and follow-up of renal function. Second, this study provides theoretical support for the potential value of early intervention for specific dyslipidemias. For example, the use of fibrates or niacin, drugs designed to improve TG and HDL cholesterol, could be considered to observe whether they can provide additional renal protective benefits on top of standard treatment. However, any treatment decision must be based on the patient’s specific circumstances and should await stronger evidence from randomized controlled trials.

Despite the numerous advantages of this study, such as adherence to rigorous systematic review guidelines, comprehensive literature search, standardized quality assessment, and meticulous statistical analysis, several limitations remain. First, all included studies were observational in design, thus preventing the establishment of a causal relationship between lipid levels and the risk of DN. Residual confounding factors (including glycemic control levels, blood pressure, baseline renal function, lifestyle factors, and medication use) may influence the reliability of the results. Second, the relatively small number of included studies for some lipid parameters may have limited the statistical power of the tests, potentially resulting in the failure to detect weak associations. Third, high heterogeneity exists among the included studies; despite employing a random-effects model and performed sensitivity analysis, heterogeneity may still affect the accuracy of the pooled effect size. Due to limitations in the original data report, it was not possible to perform adequate subgroup analysis for all potential sources of heterogeneity. Fourth, some original studies may not have adequately adjusted for all important confounding variables. Finally, due to the lack of individual-level data, conducting more comprehensive subgroup analyses by population characteristics was not feasible.

## Conclusion

This meta-analysis, a systematic review of 15 studies, confirmed that higher serum TG levels were linked to a greater risk of DN, while HDL-C showed a protective effect. TC, although positively correlated, had a weak effect, and LDL-C did not show a significant association. Despite high heterogeneity and potential publication bias, sensitivity analysis and shaving/complementation validation confirmed the robustness of the results. The risk of DN is driven by heterogeneous phenotypes, intertwined metabolic-inflammatory pathways, and early multi-mechanism damage that may originate in the renal tubules. Our work provides evidence supporting the view that different lipid components play differentiated roles in DN risk and highlights the need for future personalized risk stratification to integrate phenotypic information, inflammatory status, and early damage biomarkers.

## Data Availability

The original contributions presented in the study are included in the article/**Supplementary Material**. Further inquiries can be directed to the corresponding author.
