# Supplementary material for: Association between serum lipid levels and the risk of diabetic nephropathy: a meta-analysis
Source: Front Endocrinol (Lausanne). 2026 Apr 22;17:1783038. doi: 10.3389/fendo.2026.1783038 (PMC13143709; doi:10.3389/fendo.2026.1783038)
Supplement: Supplementary file 1 [file DataSheet1.docx]

Pubmed-1121

((((("Triglycerides"[Mesh]) OR (((Triglyceride) OR (Triacylglycerols)) OR (Triacylglycerol))) OR (((((Low-Density Lipoprotein Cholesterol) OR (LDL-C)) OR (High-Density Lipoprotein Cholesterol)) OR (HDL-C)) OR (Total Cholesterol))) AND (("Diabetic Nephropathies"[Mesh]) OR (((((((Diabetic Kidney Disease) OR (Diabetic Nephropathy)) OR (Diabetic Glomerulosclerosis)) OR (Intracapillary Glomerulosclerosis)) OR (Kimmelstiel Wilson Disease)) OR (Nodular Glomerulosclerosis)) OR (Kimmelstiel Wilson Syndrome)))) AND ((Risk) OR (Incidence))) AND ((((((Cohort) OR (Case-control)) OR (Cross-sectional)) OR (Random*)) OR (Clinical study)) OR (Clinical trial))

Embase-2772

((Triglycerides or (Triglyceride or Triacylglycerols or Triacylglycerol) or (Low-Density Lipoprotein Cholesterol or LDL-C or High-Density Lipoprotein Cholesterol or HDL-C or Total Cholesterol)) and (Diabetic Nephropathies or (Diabetic Kidney Disease or Diabetic Nephropathy or Diabetic Glomerulosclerosis or Intracapillary Glomerulosclerosis or Kimmelstiel Wilson Disease or Nodular Glomerulosclerosis or Kimmelstiel Wilson Syndrome)) and (Risk or Incidence) and (Cohort or Case-control or Cross-sectional or Random* or Clinical study or Clinical trial)).af.

Cochrane-104

((Triglycerides or (Triglyceride or Triacylglycerols or Triacylglycerol) or (Low-Density Lipoprotein Cholesterol or LDL-C or High-Density Lipoprotein Cholesterol or HDL-C or Total Cholesterol)) and (Diabetic Nephropathies or (Diabetic Kidney Disease or Diabetic Nephropathy or Diabetic Glomerulosclerosis or Intracapillary Glomerulosclerosis or Kimmelstiel Wilson Disease or Nodular Glomerulosclerosis or Kimmelstiel Wilson Syndrome)) and (Risk or Incidence) and (Cohort or Case-control or Cross-sectional or Random* or Clinical study or Clinical trial)).af.

WOS-1048

(((((Triglycerides) OR (((Triglyceride) OR (Triacylglycerols)) OR (Triacylglycerol))) OR (((((Low-Density Lipoprotein Cholesterol) OR (LDL-C)) OR (High-Density Lipoprotein Cholesterol)) OR (HDL-C)) OR (Total Cholesterol))) AND ((Diabetic Nephropathies) OR (((((((Diabetic Kidney Disease) OR (Diabetic Nephropathy)) OR (Diabetic Glomerulosclerosis)) OR (Intracapillary Glomerulosclerosis)) OR (Kimmelstiel Wilson Disease)) OR (Nodular Glomerulosclerosis)) OR (Kimmelstiel Wilson Syndrome)))) AND ((Risk) OR (Incidence))) AND ((((((Cohort) OR (Case-control)) OR (Cross-sectional)) OR (Random*)) OR (Clinical study)) OR (Clinical trial)) (Topic)
